# Cancer Imprints an Increased PARP-1 and p53-Dependent Resistance to Oxidative Stress on Lymphocytes of Patients That Later Develop Alzheimer's Disease

**DOI:** 10.3389/fnins.2018.00058

**Published:** 2018-02-08

**Authors:** Felipe Salech, Daniela P. Ponce, Carol D. SanMartín, Nicole K. Rogers, Mauricio Henríquez, Maria I. Behrens

**Affiliations:** ^1^Instituto de Ciencias Biomédicas, Facultad de Medicina, Universidad de Chile, Santiago, Chile; ^2^Centro de Investigación Clínica Avanzada (CICA), Hospital Clínico Universidad de Chile, Santiago, Chile; ^3^Departamento de Neurociencias Facultad de Medicina, Universidad de Chile, Santiago, Chile; ^4^Unidad de Geriatría, Hospital Clínico Universidad de Chile, Santiago, Chile; ^5^Departamento de Neurología y Neurocirugía, Hospital Clínico Universidad de Chile, Santiago, Chile; ^6^Clínica Alemana, Santiago, Chile

**Keywords:** cancer, AD, oxidative death resistance, lymphocytes, PARP-1, p53

## Abstract

We have proposed that a common biological mechanism deregulated in opposite directions might explain the inverse epidemiological association observed between Alzheimer's disease (AD) and cancer. Accordingly, we showed that lymphocytes from AD patients have an increased susceptibility, whereas those from survivors of a skin cancer, an increased resistance to oxidative death induced by hydrogen peroxide (H_2_O_2_), compared to healthy controls (HC). We investigated the susceptibility to H_2_O_2_-induced death of lymphocytes in survivors of any type of cancer and in cancer survivors who later developed AD (Ca&AD). We also explored the involvement of Poly [ADP-ribose] polymerase-1 (PARP-1) and p53 pathways in the process, since both are involved in the increased susceptibility to death of AD lymphocytes. Lymphocytes from 11 cancer and 13 Ca&AD patients, and 12 HC were submitted to increasing concentrations of H_2_O_2_ for 20 h. Cell death was determined by flow cytometry, in the presence or absence of PARP-1 inhibition (3-aminobenzamide, 3-ABA), or p53 inhibition (pifithrin-α) or stabilization (Nut-3). PARP-1 and p53 mRNA levels were determined by Real-Time PCR. Lymphocytes from cancer and Ca&AD patients showed increased survival compared to HC, without differences between them, opposite to the increased susceptibility to death previously shown in AD. PARP-1 inhibition provided marked protection from H_2_O_2_-induced death in the two groups of patients, significantly greater than in HC. Pharmacological inhibition of p53 increased lymphocyte survival in Ca&AD patients, contrary to the effect previously reported in HC and AD. PARP-1 and p53 mRNA levels were elevated in Ca&AD lymphocytes compared with controls. In all, these results show that cancer imprints an increased resistance to H_2_O_2_-induced death in lymphocytes that persists after AD development, and is dependent on both PARP-1 and p53. p53 inhibition showed a differential role in cancer and Ca&AD compared to HC and AD lymphocytes, that could explain the inverse susceptibility to oxidative death in cancer and AD. These results are in agreement with the hypothesis of a common biological mechanism in AD and cancer. The similar cell death susceptibility and cell death pattern observed in cancer and Ca&AD lymphocytes suggests that cancer history leaves long term effects on lymphocyte cell death susceptibility.

## Introduction

Our group and others have reported that patients with AD have a lower risk of developing cancer, and inversely, patients with a history of cancer in the past show a lower risk of developing AD. We showed an inverse association between AD and cancer in a memory and aging cohort of around 600 patients and in the Cardiovascular Health Study, a population based sample of 3,000 individuals (Roe et al., 2005, 2010). This inverse association was later replicated in several other epidemiological studies (Roe et al., 2005, 2010; Driver et al., 2012; Musicco et al., 2013; Ou et al., 2013; White et al., 2013; Frain et al., 2017). In a meta-analysis, Catala et al. found 50% reduced risk of AD in cancer survivors and a 36% lower risk of cancer in AD patients (Catalá-López et al., 2014). Furthermore, in a recent report, Frain et al. reported a reduced risk of AD in most cancer types in a US national database of 3,499,378 veterans (Frain et al., 2017). Importantly, this study also showed there was a higher risk of other age-related diseases such as, stroke, osteoarthritis, macular degeneration, and non-AD dementia, indicating that the inverse association was not simply due to ascertain bias, of non-diagnosing a serious condition when the another one is present. The inverse association was also demonstrated for skin cancers only (Roe et al., 2005; White et al., 2013), basal and squamous cell carcinomas, which represent around 50% of all cancers. Therefore, AD and cancer seem to protect each other from the development of the other pathology, however, there are a few patients having both disorders, a history of a cancer in the past that later develop AD.

This epidemiological relationship between cancer and dementia rises the interesting possibility that one or more biological mechanisms may link both disorders (Behrens et al., 2009; Tabarés-Seisdedos and Rubenstein, 2013; Driver et al., 2015). If such a mechanism can be identified, it might lead to better understanding of the pathology of these two disorders, as well as strategies to treatment development. Considering that aging is the greatest risk factor for a majority of chronic diseases, the exploration of the mechanisms associated with age-related chronic diseases might give us clues for common mechanism behind the aging processes. We have proposed that the cellular machinery controlling cell death in response to stress could be deregulated in AD and other neurodegenerative disorders leading to a more prone to death state, and in the opposite way, to a more prone to survival status in cancer (Behrens et al., 2009). Accordingly, we demonstrated that lymphocytes from AD patients show an increased susceptibility to death by H_2_O_2_ exposure, whereas those from skin cancer patients show increased survival, compared with lymphocytes from healthy control donors, without cognitive impairment, or cancer history of similar age and sex (Behrens et al., 2012). In addition, we showed that in patients with dementia, the H_2_O_2_ lymphocyte death susceptibility increased in magnitude with the severity of the disease (Ponce et al., 2014), and is also present in the early stages of the disorder, in patients with mild cognitive impairment (MCI) (Salech et al., 2017).

p53 and poly [ADP-ribose]-polymerase-1 (PARP-1) are cell repair pathways that respond to injuries produced by reactive oxygen species triggered by several damages, such as amyloid beta peptide exposure (Martire et al., 2013, 2016). Depending on the extent of the damage, p53 and/or PARP-1 can initiate DNA repair mechanisms or, on the contrary, activate programmed cell death inducing either apoptosis, or a caspase-independent, PARP-1-dependent cell death (Lakin and Jackson, 1999; Jagtap and Szabó, 2005; Green and Kroemer, 2009).

In previous studies, we showed that the susceptibility of lymphocytes to cell death induced by H_2_O_2_ in patients with cognitive impairment is dependent on both PARP-1 an p53 activity (Salech et al., 2017). In AD patients PARP-1 inhibition caused significant protection from H_2_O_2_-induced death, but did not reach control levels, suggesting that other cell death mechanisms are involved. In fact, we found that modulation of p53 in lymphocytes of healthy control subjects induced an increase in H_2_O_2_-induced death, that attained the levels seen in AD. In addition, lymphocytes from AD patients were unaffected by modulation of p53. The lack of effect of p53 modulation together with an increase in p53 mRNA levels in AD lymphocytes suggests that p53 has lost its response to modulation in this condition.

We here investigated the death of lymphocytes induced by H_2_O_2_ in survivors of any type of cancer and in survivors of cancer who later developed AD (Ca&AD). In addition, we investigated the role of PARP-1 and p53 on the regulation of the susceptibility to oxidative death in these two groups of patients and compared them with the results previously reported in AD lymphocytes (Salech et al., 2017), in order to elucidate the involvement of the cell repair machinery in the inverse susceptibility to oxidative death of lymphocytes from cancer and AD patients.

## Materials and methods

### Patients

A total of 36 individuals: 11 cancer, 13 Ca&AD patients and 12 healthy donors were recruited. All participants signed an informed consent that was approved by the Ethics Committee of the Hospital Clínico de la Universidad de Chile. Caregivers of patients with severe dementia provided the consent. Three patients from the healthy control and cancer group were analyzed in previous reports (Behrens et al., 2012; Salech et al., 2017), but donated new blood samples. For the diagnosis of AD the guidelines of Alzheimer's Association and the National Institute on Aging (McKhann et al., 2011) were followed and dementia severity was rated with the CDR (Morris, 1997). In addition, the Montreal Cognitive Assessment (MoCA) test validated by us in Spanish (Delgado et al., 2017) was applied. The maximum score for the MoCA is 30, with lower scores associated with greater cognitive deterioration. Patients in the Ca&AD group had variable degrees of cognitive impairment with CDRs ranging from 0.5 to 2 (Table 1). The types of cancer in the cancer and Ca&AD groups are shown in Table 2. The most frequent types of cancer were skin, breast and colon. The time elapsed between the cancer diagnosis and enrolment in the present study was between 3 and 20 years. Healthy controls were submitted to the same neurological and neuropsychological evaluations. Table 1 shows the demographic data of study participants.

**Table 1:** Demographic data of participants in the study.

	**Healthy controls *n* = 12**	**Cancer *n* = 11**	**Ca&AD *n* = 13**
Age, mean ± SE (range)	76.8 ± 2.7 (62–96)	71.9 ± 2.89 (60–84)	81.5 ± 2.3 (66–90)
Female sex (%)	9 (75)	9 (82)	5 (38)
Education	12.7 ± 1.3	13.6 ± 2.1	11.2 ± 1.7
CDR score	0	0	1.2 ± 0.2
CDR = 0	12	11	0
(Number of Patients)			
CDR = 0.5	0	0	5
CDR = 1	0	0	3
CDR = 2	0	0	5
MoCA test score mean ± SE	28.4 ± 0.5	28.3 ± 0.5	16.4 ± 1.7
Diabetes or Insulin Resistance (%)	7 (58)	5 (45)	4 (31)
Hypertension (%)	7 (58)	3 (27)	8 (62)
Ever smoked tobacco (%)	4 (33)	4 (36)	8 (61)

*CDR, Clinical Dementia Rating; MoCA, Montreal Cognitive Assessment*.

**Table 2:** Types of cancers in the Cancer and Ca&AD groups.

	**Cancer**	**Ca&AD**
Basal cell carcinoma	6	3
Squamous cell carcinoma	1	1
Melanoma	1	1
Breast	2 (incipient)	2
Colon	0	3
Lung	0	1
Prostate	0	2
Bladder	0	1
Lymphoma	0	1
Appendicular carcinoma	1	0
Retroperitoneal carcinoma	1	0

### Materials and equipment

Hydrogen peroxide (H_2_O_2_) was from Merck (Darmstadt, Germany), Ficoll-Hypaque™ PLUS was obtained from GE Healthcare (Little Chalfont, RU), Trizol was from Life Technology (Carlsbad, CA, USA), 3-Aminobenzamide (3-ABA), and nutlin-3 and Pifithrin-α were bought from Sigma-Aldrich (Oakville, ON, Canada), High Capacity cDNA Reverse Transcription Kit was from Thermo Fisher Scientific (Carlsbad, CA, USA), TURBO DNA- free™ Kit was from Invitrogen (Waltham, MA, USA), Brilliant III SYBER-GREEN Master Mix and MX3000P were from Agilent Technologies (La Jolla, CA, USA). Flow cytometry FACScan was from Becton Dickinson (Franklin Lakes, NJ, USA). Real-time PCR (RT-PCR) was performed in an amplification system (MX3000P, Startagene, La Joya, CA).

### Peripheral blood lymphocyte isolation and treatments

Fifteen milliliters of blood were obtained by venipuncture and peripheral blood lymphocytes were extracted by Ficoll-Hypaque density centrifugation. The extracted lymphocytes were exposed for 20 h to increasing concentrations of H_2_O_2_ (Behrens et al., 2011). To evaluate the effects of PARP-1 inhibition on H_2_O_2_-induced death, 5 mM 3-Aminobenzamide (3-ABA) was added 30 min before H_2_O_2_ exposure. The effects of p53 modulation were studied by the addition of the p53 inhibitor, Pifithrin-α (20 μM), or stabilizer, Nutlin-3 (10 μM), added 30 min before H_2_O_2_ exposure. Samples containing roughly 1 × 10^6^ cells were analyzed by flow cytometry following propidium iodide (PI) staining, in which viable (PI-negative), apoptotic (PI-positive, hypodiploid), and necrotic (PI-positive diploid) cells were distinguished (Behrens et al., 2011, 2012; Ponce et al., 2014; Salech et al., 2017).

### RNA isolation and PCR analysis

RT-PCR was carried out as previously described (Salech et al., 2017). In brief: total RNA was isolated from peripheral blood lymphocyte using Trizol reagent, and contaminating genomic DNA was removed by a DNAase digestion step with TURBO DNA- free™ Kit, and 260/280-absorbance ratio was used to assessed RNA purity. From 2 μg total RNA cDNA was synthesized using the High Capacity cDNA Reverse Transcription Kit. RT-PCR was performed using the DNA binding dye SYBR green (Brilliant II SYBER-GREEN Master Mix) in an amplification system MX3000P. The following primers were used: PARP-1: 5′-TTGAAAAAGCCCTAAAGGCTCA-3′, 5′-CTACTCGGTCCAAGATCGCC-3′. P53: 5′-AGCTTTGAGGTGCGTGTTTG-3′, 5′-TCAGCTCTCGGAACATCTCG-3′. 18S: 5′-GATATGCTCATGTGGTGTTG-3′, 5′-AATCTTCAGTCGCTCCCA-3′. Quantification was performed using the technique ΔΔCt (Pfaffl et al., 2001). Levels of PARP-1 and p53 mRNA were normalized with respect to levels of 18S mRNA. All samples were run in triplicate.

### Western blot analyses

Lymphocytes extracts were resolved by 10% SDS-PAGE, and then transferred to PDVF membranes. Blots were blocked for 1 h at room temperature in Tris-buffered saline (TBS) containing 0.2% Tween-20 and 5% fat-free milk. Overnight incubation with primary antibody against PARP-1 1:1000 (Santa Cruz, cat. number 7150) or p53 1:1,000 (Cell Signaling, cat. number 2527), was performed at 4°C. After incubation for 1.5 h with HRP-conjugated secondary antibodies, membranes were developed by enhanced chemiluminescence (Amersham Biosciences, Bath, UK). To correct for loading, membranes were stripped and blotted against β-actin 1:10,000 (Abcam, cat. number ab8227). The films were scanned and the Image J program was employed for densitometric analysis of the bands.

### Statistical analysis

Differences between the three experimental groups at each dose in lymphocyte survival, apoptosis, and necrosis were adjusted for age and sex and analyzed using general linear model in SPSS 23 with Bonferroni correction. Data from RT-PCR were analyzed using ANOVA with Bonferroni correction. Results were expressed as means ± standard error of the mean (SEM). Differences *p* ≤ 0.05 were considered statistically significant.

## Results

### Lymphocytes from cancer and Ca&AD patients showed decreased cell death susceptibility to H_2_O_2_ compared with healthy controls

**Figure 1:**
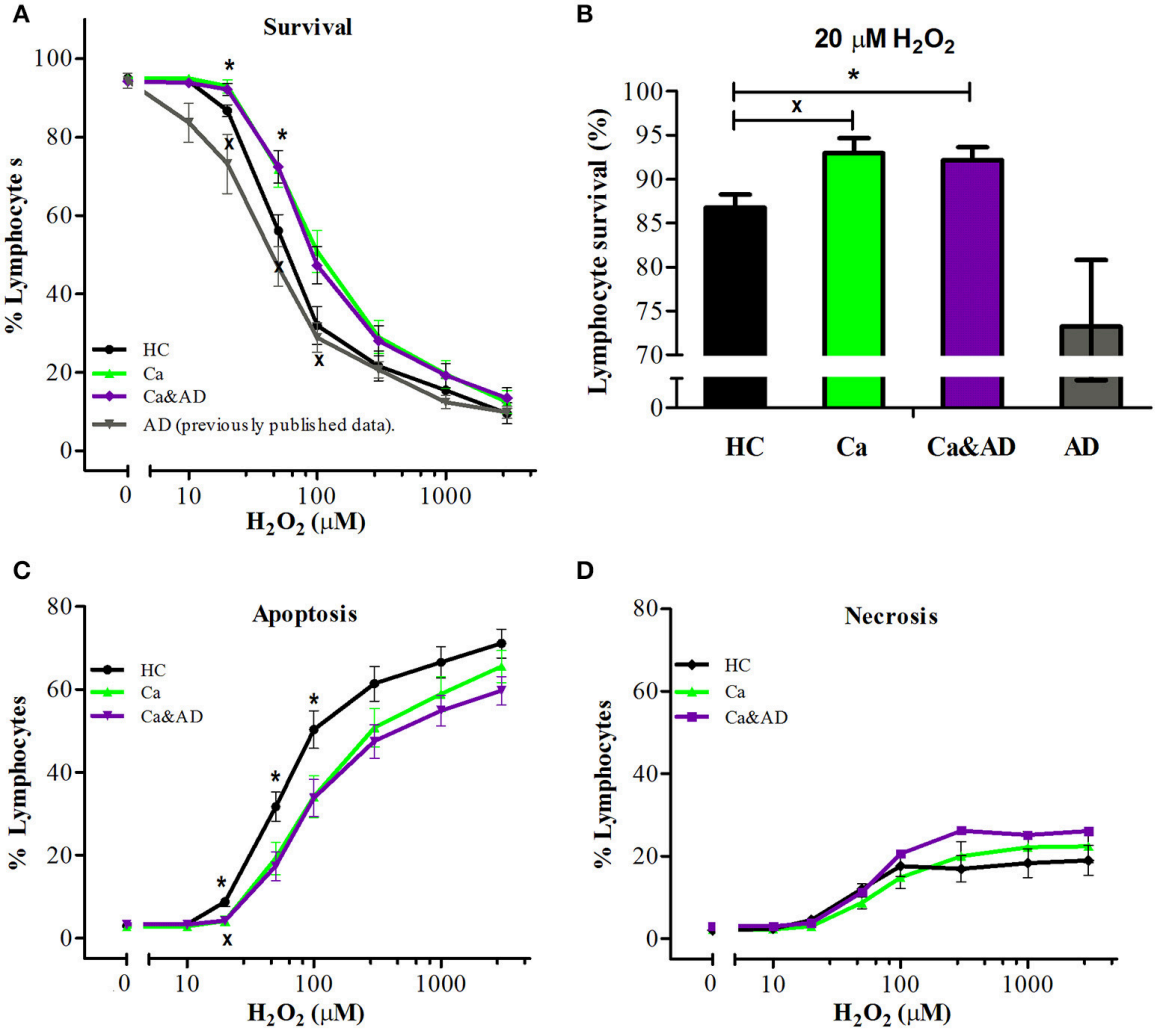
H_2_O_2_-induced death of lymphocytes from cancer, cancer & AD and healthy controls. Lymphocytes from 11 cancer patients (Ca; green symbols), 13 cancer & AD (Ca&AD; purple symbols) patients, and 12 healthy controls (HC; black symbols) were exposed to different concentrations of H_2_O_2_ for 20 h and death was determined by flow cytometry with propidium iodide staining. **(A)** Lymphocyte survival curve at increasing concentrations of H_2_O_2_; **(B)** Survival values at 20 μM H_2_O_2_; **(C,D)** apoptosis and necrosis curves, respectively, from experiments in A (%, means ± SE). The results previously reported (Salech et al., 2017) on AD lymphocytes were added in A and B for comparison. ^x^Ca vs. HC, ^*^Ca&AD vs. HC. 1 symbol: *p* < 0.05; 2 symbols: *p* < 0.005; 3 symbols: *p* < 0.0001 for all figures.

Demographic data and patient characteristics are shown in Table 1. In this study, we analyzed the susceptibility of H_2_O_2_-induced death of lymphocytes obtained from patients with a history of any type of cancer in the past and from patients who had both, a history of any cancer in the past and AD (Ca&AD), and compared them with a control group of cognitively healthy control (HC) subjects free of cancer history. As previously reported for skin cancer only patients (Behrens et al., 2012), lymphocytes from the cancer group in this study showed increased resistance to cell death induced by H_2_O_2_ exposure, compared with HC lymphocytes (Figure 1A). Lymphocytes from patients suffering from both, AD and cancer (Ca&AD) showed a very similar cell death susceptibility as the cancer group (Figure 1A). Upon treatment with 20 μM H_2_O_2_, survival values were 86.7 ± 1.5, 92.9 ± 1.7, and 92.2 ± 1.5% for HC, cancer, and Ca&AD, respectively (Figure 1B). These are compared with the AD group previously reported that showed higher susceptibility to death (73.2 ± 7.6%) (Salech et al., 2017), which we incorporated in Figures 1A,B for comparison. The increase in survival observed in cancer and Ca&AD lymphocytes compared to HC was due to reduced levels of apoptosis, without changes in necrosis (Figures 1C,D). The susceptibility to H_2_O_2_-induced cell death observed in cancer and Ca&AD groups is opposite to that described in patients with MCI and AD, where a significantly increased susceptibility to death was observed (Behrens et al., 2012; Salech et al., 2017), that was caused by increased levels of apoptosis, and also of necrosis in AD (Salech et al., 2017).

### PARP-1 inhibition protected lymphocytes from all patients, with a higher protection for Ca&AD and cancer patients over healthy controls

**Figure 2:**
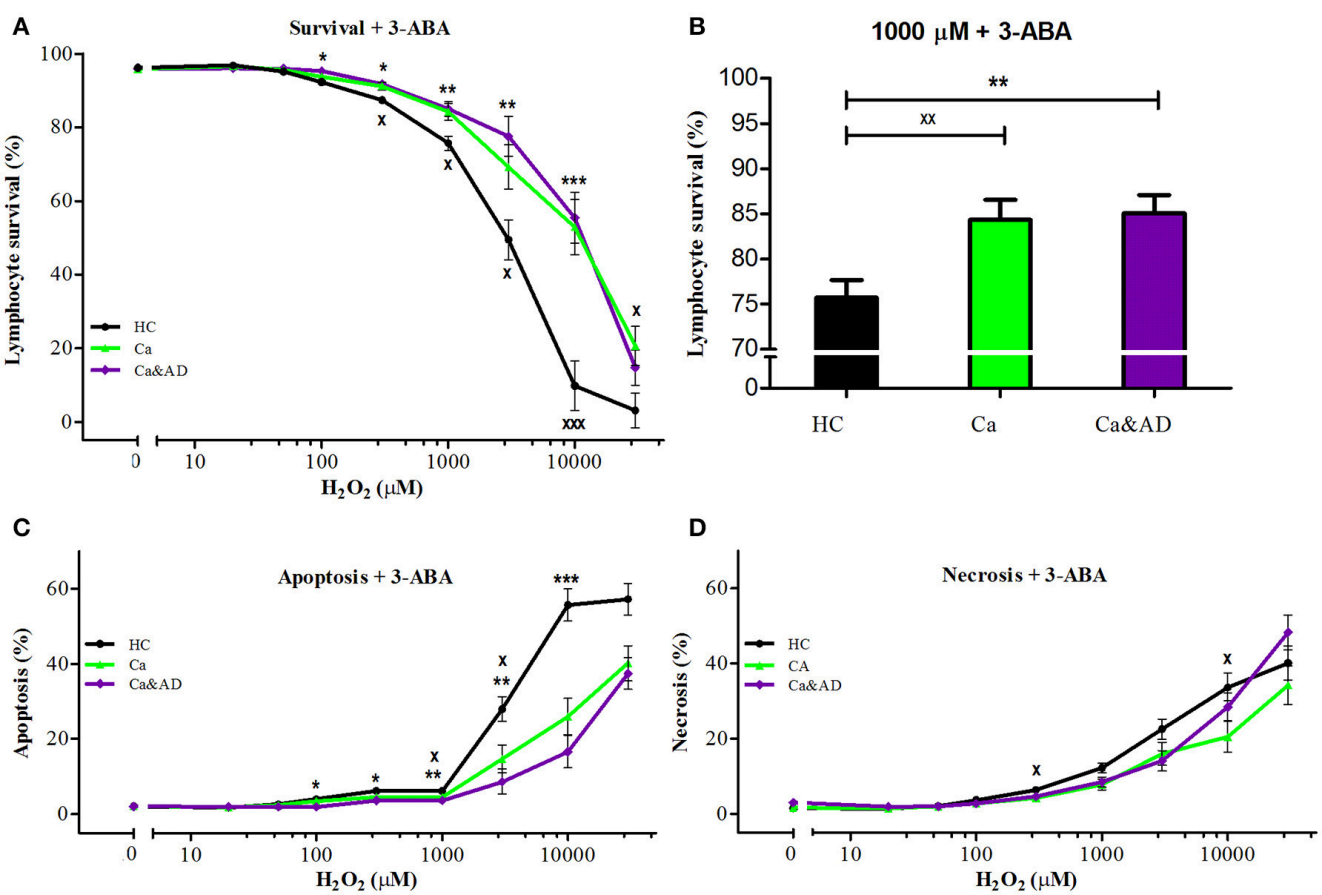
Effect of PARP-1 inhibition with 3-ABA on H_2_O_2_-induced death of lymphocytes from cancer, Ca&AD patients and healthy controls. Lymphocytes from 10 cancer patients (Ca; green symbols), 10 cancer & AD (Ca&AD); purple symbols, and 11 healthy controls (HC; black symbols) were pre-incubated with 5 mM 3ABA for 30 min and then exposed to different concentrations of H_2_O_2_ for 20 h. **(A)** Survival curves (%, means ± SE); **(B)** Survival values at 1,000 μM H_2_O_2_ (%, mean ± SE); **(C,D)** apoptosis and necrosis curves, respectively, from experiments in **(A)** (% means ± SE). Symbols and significance as in Figure 1.

PARP-1 inhibition with 3-ABA markedly protected from the death induced by H_2_O_2_ exposure in all the groups studied. Interestingly, the protection in the Ca&AD group was almost the same as in cancer patients (Figure 2A) and higher than in the HC and AD groups. At 1,000 μM H_2_O_2_ concentration, lymphocyte survival in the presence of 3-ABA was 76.7 ± 3.8, 84.2 ± 4.2, and 85.9 ± 4.1% for HC, cancer and Ca&AD, respectively, compared to 66.5 ± 5.5 in the AD group from our previous report (Figure 2B). The type of death rescued by PARP-1 inhibition with 3-ABA was mainly achieved by a decrease in apoptosis, and also by a decrease in necrosis in cancer patients (Figures 2B,C).

**Figure 3:**
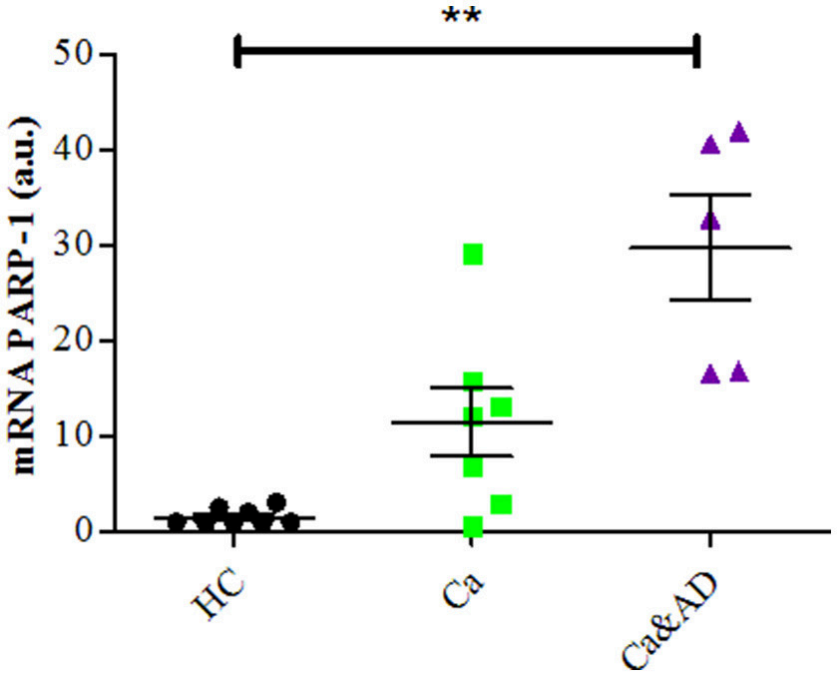
PARP-1 expression in lymphocytes. mRNA levels of PARP-1 measured by RT-PCR (means ± S.E). Healthy controls (HC, *n* = 8); cancer (Ca, *n* = 7); cancer and AD (Ca&AD, *n* = 5).

Consistent with the greater protection granted by 3-ABA, we found that Ca&AD lymphocytes had significantly higher expression levels of PARP-1 mRNA under basal conditions (Figure 3) than HC lymphocytes. Lymphocytes from cancer patients also had higher levels of PARP-1 mRNA, but that did not reach statistical significance (Figure 3), probably due to the number of patients in the study. In all, these results support the idea that in Ca&AD and cancer lymphocytes, oxidative stress induces death that is markedly PARP-1 dependent. The increased levels of PARP-1 mRNA in the cancer and Ca&AD groups might indicate a protective role of PARP-1 in these cells and thus explain the increased resistance to death in lymphocytes from patients with a history of cancer. Western blot analyses of PARP-1 protein levels did not show clear differences between the groups (Supplementary Figure 1) probably due to a great variability within groups.

### p53 in the regulation of oxidative cell death of lymphocytes from cancer and Ca&AD patients

We sought to investigate the role of p53 in the pattern of cell death in cancer and Ca&AD lymphocytes, considering the well-known role of p53 in the regulation of cell death in cancer patients and the abnormal regulation of p53 that we reported in AD lymphocytes submitted to H_2_O_2_ -dependent death.

**Figure 4:**
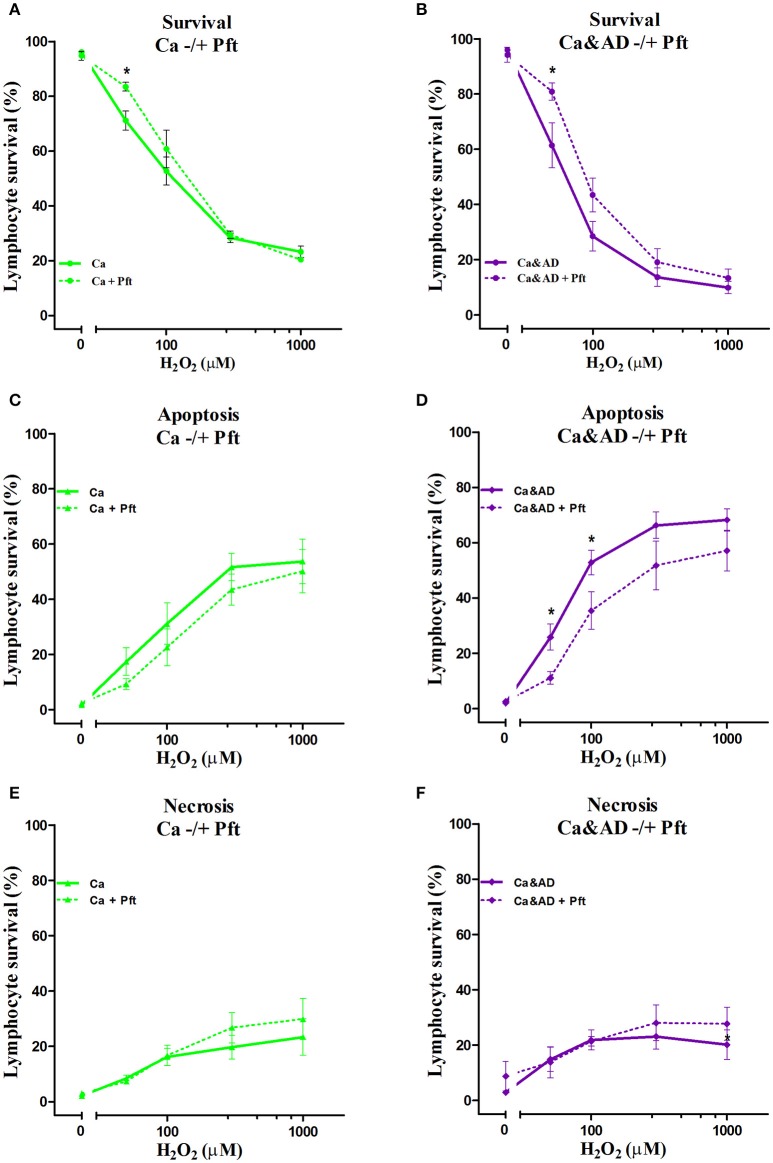
Effect of p53 modulation on H_2_O_2_-induced death of lymphocytes. Lymphocytes from 5 cancer (Ca; left panels), and 5 cancer & AD (Ca&AD; right panels) were exposed to the indicated concentrations of H_2_O_2_ in the presence or absence of 20 μM Pifithrin-α (Pft), a p53 inhibitor (short interrupted lines), applied 30 min before H_2_O_2_ incubation (means ± SE). **(A,B)** Survival curves, **(C,D)** apoptosis, **(E,F)** necrosis. Symbols and significance as in Figure 1.

In cancer and Ca&AD groups, pharmacological inhibition of p53 with pifithrin-α produced a significant increase in lymphocyte survival at 50 μM H_2_O_2_ concentration (Figures 4A,B). These results are different to the effect of p53 inhibition that we reported in lymphocytes from HC and AD patients, where a decrease in survival and a null effect, respectively, were observed (Salech et al., 2017). The analysis of the type of death induced by p53 inhibition showed that the increased survival observed in cancer and Ca&AD patients was secondary to a decrease in apoptotic death (Figures 4C,D), without modifications in necrosis. The stabilization of p53 with nutlin had no effect on H_2_O_2_-induced death, neither in cancer nor in Ca&AD lymphocytes, similar to that described in patients with AD (Salech et al., 2017) (Supplementary Figure 2).

**Figure 5:**
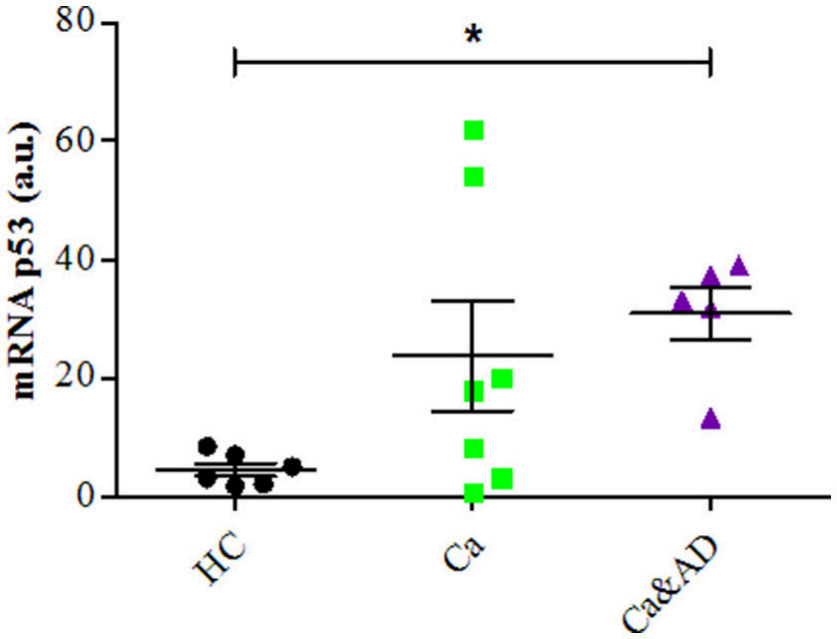
P53 expression in lymphocytes. mRNA levels of p53 measured by RT-PCR (means ± S.E). Healthy controls (HC, *n* = 8); cancer (Ca, *n* = 7); cancer and AD (Ca&AD, *n* = 5). Symbols and significance as in Figure 1.

mRNA expression of p53 determined by RT-PCR showed that consistent with the above results, the basal expression levels of p53 mRNA were significantly increased in Ca&AD lymphocytes compared to controls (Figure 5). Lymphocytes from the cancer group showed higher levels of p53 mRNA, but did not reach statistical significance (Figure 5). In all these results suggest that in addition to PARP-1, p53 also participates in the regulation of cell death susceptibility of cancer and Ca&AD patients, in a different way to that observed in lymphocytes from AD patients and healthy controls that was reported previously. As with PARP-1, western blot analyses of p53 protein levels showed great variability within the groups, without clear differences among groups (Supplementary Figure 1).

## Discussion

**Figure 6:**
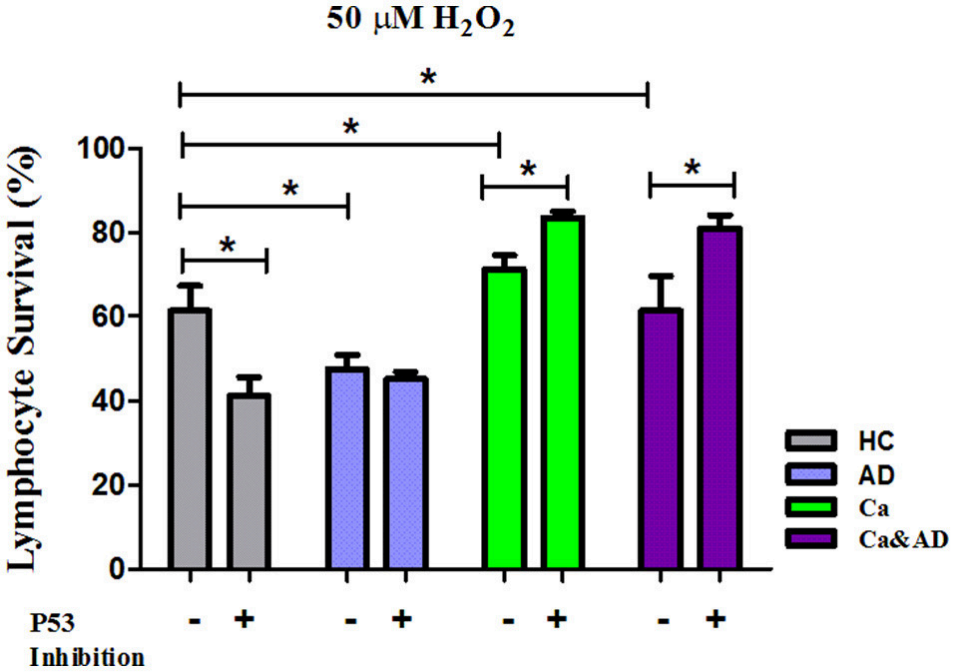
Comparison of lymphocyte survival and the effect of p53 inhibition among the different groups. Lymphocytes survival at 20 μM H_2_O_2_ in the presence or absence of the p53 inhibitor Pifithrin-α (Pft) (20 μM) in cancer and Ca&AD groups; the data for AD and healthy control groups are from our previous report (Salech et al., 2017), shown here for comparison (means ± SE).

In this study, we show that patients with a past history of cancer of any type and those suffering from the two disorders, a history of cancer who developed AD (Ca&AD), showed increased resistance to H_2_O_2_-induced death compared with HC. This result is opposite to that reported in AD lymphocytes which showed higher susceptibility to H_2_O_2_-induced death (Salech et al., 2017). The higher resistance to death observed in lymphocytes from cancer an Ca&AD patients confirms our previous study showing an increased resistance in a skin cancer only group of patients (Behrens et al., 2012). Therefore, a higher resistance to cell death could be a more generalized effect in all cancer types, in agreement with the inverse association demonstrated in epidemiological studies between AD and all types of cancers (Roe et al., 2005, 2010; Musicco et al., 2013; Ou et al., 2013). We also show that the death induced by H_2_O_2_ in cancer and Ca&AD lymphocytes is modulated by both PARP-1 and p53. These same pathways are involved in the H_2_O_2_-induced death of lymphocytes from HC and AD patients, as we previously showed. However interestingly, the inhibition of p53 in the cancer and Ca&AD groups of patients in this study induced and increase in survival, which is opposite to the effect of p53 inhibition in lymphocytes from AD patients and HC subjects in our previous report (Figure 6) (Salech et al., 2017). These results suggest that a deregulation of the p53 pathway may be involved in the mechanism of the inverse association seen between AD and cancer.

Oxidative cell death is involved in several chronic disorders (Patlevič et al., 2016; Di Domenico et al., 2017; Sack et al., 2017) and has been suggested as one important contributor to cellular aging (Miyoshi et al., 2006). Reactive oxygen species generated by oxidative stress due to H_2_O_2_, activate the cellular machinery to avoid damage to the DNA and other cellular components. p53 and PARP-1 are both activated upon oxidative stress. When the level of damage is low, p53 and PARP-1 facilitate DNA repair mechanisms and have been called guardians of the genome. However, upon intense damage, they promote cell death. p53 is a transcription factor that upon stress induces either, cell cycle arrest, senescence or apoptosis (Lakin and Jackson, 1999; Green and Kroemer, 2009). PARP-1 is a nuclear enzyme that participates in DNA repair, but under severe damage, consumes nicotinamide adenine dinucleotide (NAD^+^), depleting the cell of energy and therefore leading to a caspase-independent cell death (Lakin and Jackson, 1999; Jagtap and Szabó, 2005). In a neuroblastoma cell line, PARP-1 has an important role in the molecular mechanism of amyloid beta peptide damage, inducing mitochondrial dysfunction and cell death by a 60% increase in its activity (Martire et al., 2016). This PARP-1-dependent, caspase-independent type of cell death has been named parthanatos (Wang et al., 2009; Fatokun et al., 2014; Dawson and Dawson, 2017). We have reported previously that the death of human lymphocytes induced by H_2_O_2_ is very markedly protected by PARP-1 inhibition, and is not accompanied by changes in caspase activity, suggesting that it belongs to this type of death (Behrens et al., 2011).

We here show that PARP-1 has also a preponderant role in the death of lymphocytes induced by H_2_O_2_ in the cancer and Ca&AD groups. In previous reports (Behrens et al., 2012) we showed that PARP-1 inhibition had a higher protection in lymphocytes from skin cancer patients compared to those of AD patients, with control lymphocytes showing intermediate values. Here we reproduce these results in lymphocytes from cancer patients including different types of cancer. In addition, we show that Ca&AD lymphocytes showed levels of protection very similar to the cancer group. This result together with the higher levels of PARP-1 mRNA expression observed in Ca&AD lymphocytes (and also a non-significant increase in cancer lymphocytes), suggests that having had a cancer in the past might leave a trace of higher levels of PARP-1 that protects cells from oxidative death. However, at the protein level, we didn't see clear differences between the groups because of great variability within groups. In addition, it is interesting to remark that we also found an increase in PARP-1 mRNA levels in MCI lymphocytes, but not in AD (Salech et al., 2017), in support of the idea of a protective role of PARP-1, since, the increase in mRNA of PARP-1 would be present at initial stages of cognitive deterioration, but decreases as the disease progresses to AD.

In addition to the important role of PARP-1 in lymphocyte oxidative death, p53 also participates in the death of cancer and Ca&AD groups, since inhibition of p53 induced an increase in survival in cancer and Ca&AD groups. These results are different from those seen in control lymphocytes where p53 inhibition and also p53 stabilization induced an increase in the H_2_O_2_-induced death, suggesting that p53 is involved in maintaining an equilibrium in the regulation of cell survival (Salech et al., 2017). The results shown here are also different from those of AD and MCI lymphocytes, where p53 inhibition had no effect on H_2_O_2_-induced death, suggesting that cells of AD and MCI patients have lost their regulation of cell survival by p53. Therefore, a differential role of p53 may explain the inverse susceptibility to oxidative death of lymphocytes from cancer and AD patients. These results are consistent with the concept of a common biological mechanisms involved in cancer and AD pathogenesis. mRNA expression of p53 was higher in the Ca&AD group (with a non-significant increase in the cancer group). As with PARP-1 the protein levels of p53 measured with Western blot were very variable within groups which precluded further conclusions.

The Ca&AD group had a higher representation of men than the other groups in which there was a predominance of women (Table 1). The explanation for this difference might be related to the small number of patients. However, a higher prevalence of chronic diseases, including cardiac arrhythmia, chronic obstructive pulmonary disease and cancer, has been reported in male patients with AD that might also explain the higher male/female ratio in the Ca&AD group (Gambassi et al., 1999; Buchanan et al., 2004).

The idea that different aging related diseases share common biological mechanism is gaining relevance with the development of geroscience, an interdisciplinary field that aims to understand the relationship between aging and age-related diseases under the hypothesis that chronic disease and aging share common molecular mechanisms (Burch et al., 2014). There is much evidence from animal models that support this hypothesis, but the evidence from models based in human data is sparse (Kirkland, Cold Spring Harb Perspect Med 2015). An altered adaptation to stress, the regulation of cell senescence, and mitochondrial dysfunction are well-recognized aging hallmarks (López-Otín et al., 2013; Kennedy et al., 2014), and the p53 pathway participates in the regulation of all of them (López-Otín et al., 2013; Rufini et al., 2013; Fang et al., 2016). So, our results contribute to this field with evidence showing that an aging-related pathway (p53) regulates an important pathological process such as cell death, in two different age-related diseases (AD and cancer), in an experimental set-up based in older humans.

Patients with Ca&AD are a very interesting study group, because as they have a past history of cancer and an actual AD, we expected they would show characteristics more similar to AD patients. However, they showed features more similar to cancer patients, such as a decreased susceptibility to H_2_O_2_-induced death compared with lymphocytes from HC, and a potent protective effect of PARP-1 or p53 pharmacological inhibition. These results are also in agreement with the idea that cancer history might leave long term effects in lymphocytes cell death susceptibility, that may be involved in the AD reduced incidence seen in cancer patients.

Finally, the good correlation between the different characteristics of the lymphocyte cell death susceptibility showed by our results also adds to the increasing evidence that peripheral tissues show changes in cancer and neurodegenerative disorders, which represent a much simple tissue to investigate the mechanisms of disease in humans.

## Author contributions

FS: substantial contributions to the conception and design of the work; acquisition, analysis, and interpretation of data; drafting and revising the work for important intellectual content; final approval of the version to be published; DP: substantial contributions to the conception or design of the work; acquisition, analysis, and interpretation of data; drafting the work and revising it critically for important intellectual content; final approval of the version to be published; CS: substantial contribution to design of the work; acquisition, analysis, and interpretation of data; drafting and revising the work; final approval of the version to be published; NR: acquisition and analysis of data for the work; final approval of the version to be published; MH: substantial contributions to the design of the work, analysis, and interpretation of data; final approval of the version to be published; MB: substantial contributions to the conception and design of the work; the acquisition, analysis, and interpretation of data; drafting the work and revising it critically for important intellectual content; final approval of the version to be published; agreement to be accountable for all aspects the work.

### Conflict of interest statement

The authors declare that the research was conducted in the absence of any commercial or financial relationships that could be construed as a potential conflict of interest.

## Supplementary material

Supplementary Figure 1 Protein levels of PARP-1 and p53 measured by Western blot in human lymphocytes. **(A)** PARP-1 protein blot and protein levels standardized by b-actin in Healthy controls (HC, *n* = 3); cancer (Ca, *n* = 3); cancer and AD (Ca&AD, *n* = 3), and AD (AD, *n* = 3). No significant differences were found among groups, because of great variability. **(B)** p53 protein blot and protein levels standardized by b-actin in Healthy controls (HC, *n* = 2); cancer (Ca, *n* = 3); cancer and AD (Ca&AD, *n* = 3) and AD (AD, *n* = 3). No significant differences were found among groups.

Supplementary Figure 2 Effect of p53 modulation on H_2_O_2_-induced death of lymphocytes. Lymphocytes from 14 cancer (Ca; left panels), and 10 cancer & AD (Ca&AD; right panels) were exposed to the indicated concentrations of H_2_O_2_ in the presence or absence of 10 μM Nutlin (Nut), a p53 stabilizer (short interrupted lines), applied 30 min before H_2_O_2_ incubation (means ± SE). **(A,B)** Survival curves, **(C,D)** apoptosis, **(E,F)** necrosis. No significant differences were found among groups. Symbols and significance as in Figure 1.
